# Real-Time Auditory Feedback–Induced Adaptation to Walking Among Seniors Using the Heel2Toe Sensor: Proof-of-Concept Study

**DOI:** 10.2196/13889

**Published:** 2019-12-11

**Authors:** Kedar KV Mate, Ahmed Abou-Sharkh, José A Morais, Nancy E Mayo

**Affiliations:** 1 McGill University Health Centre Research Institute Montreal, QC Canada; 2 School of Physical and Occupational Therapy McGill University Montreal, QC Canada

**Keywords:** angular velocity, auditory feedback, walking, older adults

## Abstract

**Background:**

Evidence shows that gait training in older adults is effective in improving the gait pattern, but the effects abate with cessation of training. During gait training, therapists use a number of verbal and visual cues to place the heel first when stepping. This simple strategy changes posture from stooped to upright, lengthens the stride, stimulates pelvic and trunk rotation, and facilitates arm swing. These principles guided the development of the Heel2Toe sensor that provides real-time auditory feedback for each good step, in which the heel strikes first.

**Objective:**

This feasibility study aimed (1) to contribute evidence toward the feasibility and efficacy potential for home use of the Heel2Toe sensor that provides real-time feedback and (2) to estimate changes in gait parameters after five training sessions using the sensor.

**Methods:**

A pre-post study included 5 training sessions over 2 weeks in the community on a purposive sample of six seniors. Proportion of good steps, angular velocity (AV) at each step, and cadence over a 2- minute period were assessed as was usability and experience.

**Results:**

All gait parameters, proportion of good steps, AV, and duration of walking bouts improved. The coefficient of variation of AV decreased, indicating consistency of stepping.

**Conclusions:**

Efficacy potential and feasibility of the Heel2Toe sensor were demonstrated.

## Introduction

### Background

Aging renders people vulnerable to gait deviations that impair efficient walking and limits the likelihood of achieving walking targets for health promotion. Physical activity guidelines for seniors recommend a target of 150 minutes of moderate intensity exercise accumulated over 1 week in bouts of 10 minutes [1]. Walking is the most practical exercise as it requires no equipment, or no specialized environment [2], and produces many physical and cognitive health benefits from the mental stimulation of exploring new avenues or neighborhoods [3]. Maintaining a level of physical activity is also critical to prevent secondary health conditions including cardiovascular disease, osteoporosis, obesity, and diabetes [4]. Despite capacity to walk at a health-promoting pace when tested clinically, it is rare for the North American seniors to do this in the real world for more than a few minutes a day [5,6]. It is hard to sustain walking without the capacity for an optimal stepping pattern indicating that quality drives quantity.

Reasons for failure to use walking capacity to achieve health-promoting walking targets include fear of falling or age-related gait abnormalities [6]. These are known to cascade into a slow, unstable, shuffling pattern that increases the work of walking, fatigue, and risk of falls and hip fracture [7]. There is a considerable evidence on how to improve seniors’ gait [8,9], and evidence shows that gait training is effective in improving gait pattern [10] but effects abate with cessation of training [11]. Hence, gait training alone will not translate into the sustained behavioral change needed for physical activity guidelines to be met.

During gait training, therapists use many of verbal and visual cues to emphasize stepping with heel first. This simple strategy changes posture from stooped to upright, lengthens the stride, stimulates pelvic and trunk rotation, and facilitates arm swing [12]. However, once verbal cueing ceases, patients frequently revert to an inefficient foot-flat gait.

For walking to become more normalized, people must relearn the motor sequences of good walking and develop the needed adjuncts to efficient walking: flexibility, strength, power, core stability, balance, and trunk rotation indicated by arm swing. Therapy can work on the adjuncts, but motor learning requires instruction, practice, and feedback. The 2013 review by Sigrist et al [13] frames motor learning as a lasting change of motor performance caused by training in which the parameters of a *motor program* are developed, and there is a gradual reduction of the variability in the newly developed motor program stimulated by sensory feedback loops. The phenomenon underlying motor learning is neural plasticity [14]. A 2014 review of this topic indicates that motor learning takes place with active practice of a skill and that this activity-dependent neural plasticity can be induced by both lengthy-extensive and brief-intensive practice [14]. The literature supports the benefit of augmented or extrinsic feedback for motor learning [14]. In particular, sonification for correct movement sequences has been shown to enhance motor learning in athletes [13,15].

It is well established that knowledge of performance is strongly associated with skill acquisition and motor learning compared with knowledge of results [16,17]. Technology is poised to provide this feedback. For walking, there are emerging technologies that use footwear-based gait monitoring systems [18]. None of the reviewed technologies provided real-time feedback, and all needed considerable data processing to produce usable information on walking performance. There is evidence that gait can be modified in response to real-time auditory feedback, but currently, no technology provides this type of feedback.

These principles guided the development of the Heel2Toe sensor, a biofeedback device that provides auditory feedback for each *good* step, in which the heel strikes first. The aim of this project was to bridge this *feedback* gap that exists outside clinical settings and equip seniors to practice correct gait at convenience. The hardware and algorithm underlying generation of auditory feedback from the Heel2Toe sensor are described elsewhere [19,20]. Briefly, Heel2Toe is a modification of an off-the-shelf device from the Shimmer Motion Development Kit. The sensor is a combination of three-axis accelerometer, a three-axis gyroscope, and a microcontroller. The algorithm detects the rate of angular velocity (AV) in sagittal plane at the ankle joint and provides an auditory *beep* when the rate of foot deceleration after heel strike crosses a threshold. Pilot work on Heel2Toe has demonstrated that it is highly accurate to detect *good* steps in clinical setting [19,20]. Starting the gait cycle with a strong heel strike lengthens the stride and changes posture from stooped to upright [12,21], indicating the value of focusing on AV as a treatment target.

### Objective

The aim of this study was to contribute evidence toward the feasibility and efficacy potential for home use of theHeel2Toe sensor that provides real-time feedback for good heel strike when walking. Specifically, the objectives were (1) to identify the extent of the immediate response to the feedback, carry over when walking without feedback, and peak response to feedback and (2) to identify pleasures and challenges in using the feedback sensor.

## Methods

### Study Design

A pre-post study design, with five sessions of training over 2 weeks, was employed to estimate the efficacy potential and identify feasibility issues of the Heel2Toe sensor when deployed for walking in the community.

### Participants

A purposive sample of six people, four women and two men, over the age of 70 years, was identified from geriatric services at the Montreal General Hospital from September to October 2017. Participants were identified by a geriatrician or other health care professionals and included if they reported no limitation in walking without an aid and no cognitive impairments. The participants were selected to have a range of walking capacity from very limited to functional. Ethical approval was obtained from the Ethics Review Board of McGill University, Health Centre Research Institute.

### Measures

Participants were assessed on physical performance tests and self-report measures. Physical performance tests included gait speed, 30-second chair stand, and 2-minute walk without and with auditory feedback. The self-report questionnaires included single item on perceived walking speed, lower extremity function scale (LEFS), life space mobility scale, and activity-specific balance confidence scale. LEFS scoring is based on fit to the Rasch Model, and therefore, not all items have to be administered to derive a legitimate total score [22]. Posttraining outcomes additionally included questions about the system usability and a semistructured interview on challenges and pleasures of using the Heel2Toe sensor. The interview was conducted separately with each participant.

### Intervention

**Figure 1:**
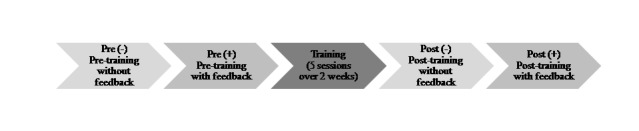
Flowchart of the study method and assessment time points.

The intervention involved a therapist visiting a participant’s residence to provide walking training with the Heel2Toe sensor, for five sessions over 2 weeks (Figure 1). The training involved walking in the participant’s neighborhood with the sensor. Care was taken that they walked on an obstacle-free path. The duration of the training was determined by the participants themselves based on interest and tolerance. On each training day, participants were instructed to walk for at least 15 minutes with the sensor at a comfortable pace and taking rests when needed. The training was accompanied with home exercises targeting flexibility and strength at ankle, knee, and hip joints with a particular focus on core strength and trunk rotation. At the end of the training, a semistructured interview was conducted with all participants.

### Analysis

The gait signals recorded with the Heel2Toe sensor were analyzed using MATLAB (MATLAB and Statistics Toolbox Release 2017b, The MathWorks, Inc, Natick, Massachusetts). The gait parameters extracted for each person over the entire walking period were proportion of good steps (%), total walking time (seconds), and average cadence (steps per minute). AV (degrees per second) in sagittal plane at ankle joint during heel strike was extracted for each step and averaged over the walking duration yielding mean, SD, and coefficient of variation (an indicator of consistency of stepping).

## Results

Table 1 shows the characteristics and level of physical activity of the participants before the training. There were two men and four women (age range: 73-87 years). The results are presented as single subjects, as it is not meaningful to aggregate data across six participants.

The score on 30-second chair rise test ranged from 0 to 12 for the six participants. Of the six participants, four exceeded their 30-second chair rise normative value. The self-reported walking speed ranged from normal to very slow walking. LEFS scale is a self-report questionnaire on difficulties with activities of daily living related to lower limb problems. The maximum score of LEFS is 32, with lower scores indicating difficulty in activities. The LEFS scores ranged from 11 (participant A) to 26 (participants D and F). Life space mobility scores ranged from 48 (participant A) to 126 (participant E) out of a total 140 days. A score of 28 days indicates no movement outside of home in the past 28 days, and a score of at least 56 indicates mobility outside of house but within the yard, porch, or apartment building.

Table 2 shows an immediate response (pre without and with feedback) and carry over effects after five training sessions (pre to post) to auditory feedback on proportion of good steps, AV, cadence, and coefficient of variation.

Posttraining gait was assessed within 1 week of the last training session. Important gains are indicated by the values in italics in Table 2. Participant A showed an immediate response to feedback producing, at first exposure, 0 good steps without any feedback and 56% good steps with feedback. This immediate response did not impact cadence, but AV showed a large effect (−48°/sec to −102°/sec). However, the coefficient of variation of AV was very large and remained so throughout. Posttraining, participant A showed some carry-over effect, as posttraining good steps without feedback changed from 0% to 29%.

Participant B produced almost twice the proportion of good steps with an increase in AV and no loss in cadence. A total of four of six participants showed only a small increase in the proportion of good steps with feedback, but all already had a high proportion (80%) of good steps without feedback. Nevertheless, they showed an improvement in AV while maintaining cadence. Overall, five of six participants showed important gains on gait parameters. The one person who did not was very good at study entry.

**Table 1:** Characteristics and physical activity level of the participants at study entry.

Characteristics/activity level			Identification					
			A (Man)	B (Woman)	C (Woman)	D (Woman)	E (Woman)	F (Man)
Age (years)			80	73	87	83	85	86
**Physical performance tests**								
	Self-report walking speed^a^		Very slow	Stroll	Stroll	Normal	Very slow	Normal
	30-second sit to stand (n)		0	12	7	9	10	12
	Age norm (n)^b^		10	10	8	8	8	8
**Self-report questionnaires**								
	**LEFS** ^c^ **(scored from 0=extreme difficulty to 4=no difficulty)**							
		Walking a mile	1	2	0	4	0	3
		Running on even ground	0	0	2	1	0	2
		Squatting	2	2	1	3	4	3
		Standing for 1 hour	2	1	0	4	2	3
		Climbing 10 stairs	2	3	3	4	4	3
		Heavy household activities	0	1	1	4	2	4
		Getting in and out of bath	3	0	4	2	4	4
		Light household activities	2	2	4	4	4	4
		Total score (0-32)	12	11	15	26	20	26
	**Life space mobility (number of days out of the past 28 days)^d^**							
		Other rooms besides the bedroom	28	28	28	28	28	28
		Areas outside home	6	28	28	28	28	15
		Places in neighborhood	6	28	15	28	28	5
		Places outside neighborhood within town	6	28	10	28	28	2
		Places outside town	2	2	2	10	14	2
		Total days (max 140 days)	48	114	83	122	126	52
	**Activity Specific Balance Confidence Scale (0%=no confidence to 100%=full confidence)**							
		Walk around the house	90	60	80	100	95	100
		Walk across a parking lot	90	50	100	100	100	100
		Walk in a crowded mall	95	50	75	90	100	100

^a^Self-reported walking speed: unable to walk, very slow, stroll at an easy pace, normal speed, fairly brisk, fast.

^b^As per Bennell et al [23].

^c^LEFS: Lower Extremity Function Scale (selected items).

^d^A score of 28 days indicates no movement outside of the home in the past 28 days; score of 56 indicates mobility outside of the house but within the yard, porch, or apartment building; score of 84 indicates going to places in neighborhood; score of 112 indicates going to places outside the neighborhood but within town; and score of 140 indicates going to places outside the town.

**Table 2:** Immediate response and carry-over effect after five training sessions with the Heel2Toe sensor on gait parameters measured without feedback (values in italics indicate clinically important changes after five days of training based on a change of ≥10%).

Outcomes and assessment			Participants’ identification					
Gait parameters and time points		Feedback	A	B	C	D	E	F
**Good steps (%) (closer to 100 is better)**								
	Pre	−^a^	0	43	80	84	92	93
	Pre	+^b^	*56*	*82*	83	97	92	99
	Post	−	29	*80*	*89*	*97*	95	99
	Post	+	*66*	*90*	*92*	*94*	93	100
**Cadence (steps/min) (closer to 100 is better)**								
	Pre	−	70	95	97	110	113	96
	Pre	+	69	102	95	95	110	95
	Post	−	77	104	100	121	105	110
	Post	+	*84*	96	99	122	111	109
**Heel strike angular velocity (°/second) (typical values are −300 to −500; the more negative, the better) [24]**								
	Pre	−	−48	−97	−147	−145	−165	−163
	Pre	+	−*102*	−128	−157	−186	−173	−213
	Post	−	−*80*	−126	−163	−*227*	−176	−*250*
	Post	+	−*102*	−*147*	−159	−*208*	−173	−*263*
**Gait regularity (angular velocity coefficient of variation; <10%) [25]**								
	Pre	−	59	39	40	31	24	24
	Pre	+	41	33	39	*17*	23	14
	Post	−	52	33	*24*	*20*	17	*11*
	Post	+	50	21	*21*	*24*	22	*10*

^a^No feedback (auditory beep was absent).

^b^Feedback provided (auditory beep was present).

**Figure 2:**
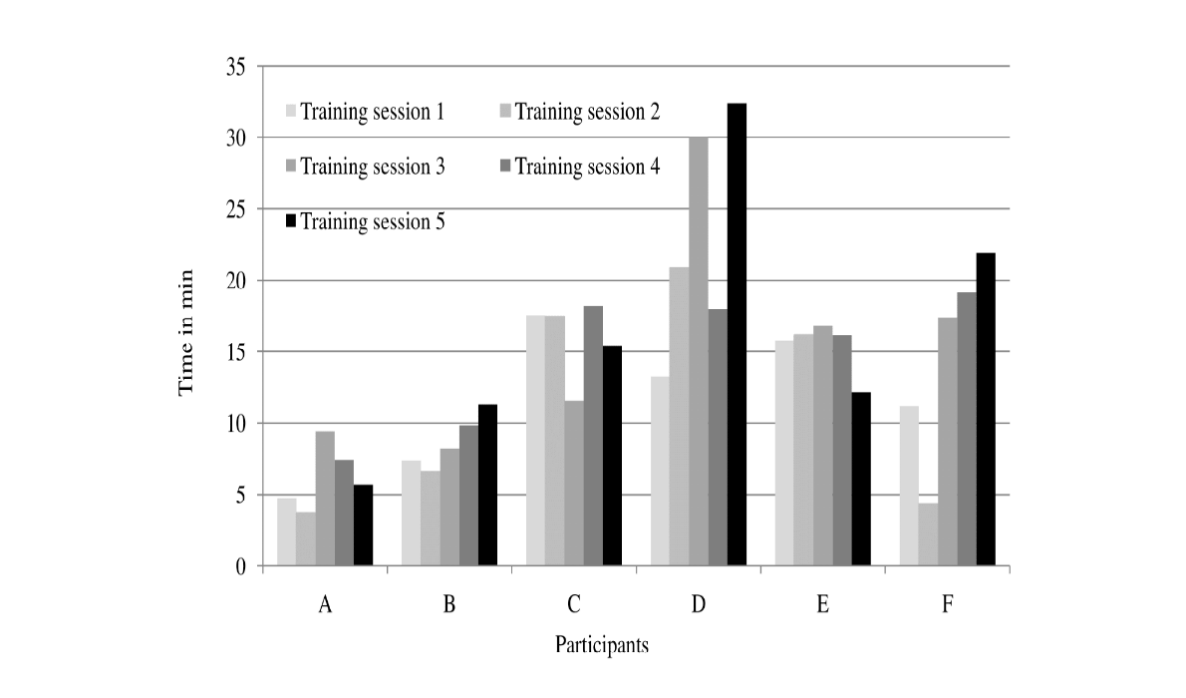
Time (minutes) spent walking with the sensor during each training day.

Figure 2 shows the duration (minutes) of intervention time over 5 training days. To illustrate, participant A, who was the most disabled, walked with the sensor for 4.5, 3.7, 9.4, 7.4, and 5.6 minutes on days 1 through 5, respectively. However participant D, who walked for about 12 minutes on day 1, had 2 days in which she walked for 30 min. All participants increased the time spent walking with the sensor over the intervention period. Out of the 30 intervention days, continuous walking bouts of 10 minutes or more were observed on 21 of the intervention days.

The information collected on system usability and on challenges and pleasures of using the Heel2Toe sensor was helpful in identifying areas for improvement. The results from the System Usability Scale are given in Table 3.

Only 8 of the original 10 questions were applicable, as 1 question was not understood and 1 question referred to their impression of how other people would be able to use the sensor. Overall, 38 item responses were available: 25 favorable, 4 neutral, and 9 unfavorable. No one feature was consistently rated *unusable,* but one issue raised concerned the intrusiveness of the sound while walking in public. This issue can be easily resolved by using earphones. The question on confidence was inconsistently answered because the trainer was always present during these training sessions.

The aim of semistructured interviews was to capture the experiences of the participants while walking with the Heel2Toe sensor in the community and recommendations for subsequent sensor development and upgrading. All participants expressed that the sensor was enjoyable, stimulating, beneficial, and easy to use while training outside the home. The participants had a few recommendations to make the sensor more user friendly. First, clipping the sensor to the shoe was recommended over a strap to accommodate older adults with back pain and limited trunk mobility. The clip also offers flexibility of use with any shoe. Second, the sensor should be available to connect via an iPad that offers a larger display for an app. Third, the sensor and app combination should be affordable and accompanied by an exercise manual.

**Table 3:** Item scores on the System Usability Scale.

Item (8/10 original questions)^a^		Response scores across participants					
		A	B	C	D	E	F
**Higher is better**							
	Use it frequently	4	3	5	—^b^	5	4
	Easy to use	4	1	1	—	5	5
	Functions integrated	5	1	3	—	1	—
	Confidence in using	5	1	—	—	1	4
**Lower is better**							
	Too complex	1	1	1	—	1	1
	Need assistance to use	1	1	1	—	1	1
	Cumbersome or awkward	1	1	5	—	2	1
	Need to learn a lot before using	1	5	1	—	2	5

^a^Two questions were omitted because of understanding (too much inconsistency with sensor) and applying what other people might think (I would imagine most people would learn to use this very quickly). Of the 18-item responses for the four questions where higher is better, 10 were at the two highest agreement levels and 6 were at the lowest levels. Of the 20-item responses for four items where lower is better, 15 were at the best level and 3 were at the poorest level.

^b^Not available.

## Discussion

### Principal Findings

We found that the Heel2Toe sensor was feasible to use in the community setting with older adults and that they improved on gait quality after the planned five training sessions, averaging 73 minutes (range: 43-114 minutes) in total. The proportion of good steps and AV improved without any detriment to cadence. All six participants showed longer duration of time spent in walking from the initial training days. However, the most dramatic effect was seen for duration of walking bouts which frequently exceeded 10 minutes (Figure 2) such that most (five of six; Table 2) participants would now be capable of meeting the Canadian Physical Activity Guidelines of 150 minutes of moderate to vigorous activity (required walking cadence ≥100 steps per minute) per week in bouts of 10 min.

Posttraining, five of six participants showed a reduction in the coefficient of variation of AV, a parameter indicating inconsistency of stepping pattern. Before training, the coefficient of variation ranged from 23% to 59%. Previous studies have shown a higher coefficient of variation in step width, and stance and stride time among older adults is associated with increased occurrence of falls [26-28], with the suggestion that a treatment target is to reduce the coefficient of variation with exercise interventions. After 5 days of training, the range was 9% to 49%.

We purposely chose a sample of people diverse in physical function. In all, two people were quite frail (A and B). Participant A was severely limited in mobility (Table 1), yet he improved on the proportion of good steps and degree and consistency of AV (Table 2 and Figure 2). Participant B also improved on these parameters. The most functional walker, participant D, showed no change as she was high on all parameters but enjoyed the experience of the sensor and could see how it would prevent deterioration. In a definitive trial, these data can be used to optimally select people for intervention.

How did the sensor achieve these outcomes? One hypothesis is that the auditory feedback acts as a positive reinforcement to a rhythmic stepping pattern. With symmetrical walking, each good step produces a *beat* that is repeated with periodicity. To produce the rhythmic pattern (the *beat*), the participants modified their stepping pattern to maintain the rhythm. In the long run, auditory cues could enhance cortical motor excitability. This has previously been studied with upper limb movements and walking tasks that required persons synchronizing to an external auditory cue [29]. The underlying basis of auditory motor synchronization is that brain poses anticipatory tendency for a rhythm, and this anticipation guides subsequent movements [29].

The Heel2Toe sensor provides direct positive auditory feedback, which could be perceived as rewarding stimulating neural plasticity and increasing the pleasure in walking, stimulating behavior change. Ultimately, the aim is to improve health-promoting walking rather than just functional walking, so that older people can derive pleasure and health benefit from walking. The sensor is not designed to be worn all the time but to be worn to practice optimal walking with the aim that this would carry over into other walking activities. As it is linked to a smartphone and the sensor is very small (size of a matchbox), it could be worn for longer periods of time.

Fear of falling and age- or illness-related changes co-occur in most seniors and can induce an inefficient and dangerous gait pattern [30,31]. To normalize walking, people must relearn motor sequences of good walking and develop needed adjuncts to efficient walking: flexibility, strength, power, core stability, balance, and arm swing. Therapy targets adjuncts but motor learning requires instruction, practice, and feedback. Motor learning is framed as a lasting change of performance occurring with training in which parameters of a *motor program* are developed and consolidated. Early on, formation of the motor program of the *to-be-learned task* can occur rapidly but demands high levels of attention. Later, the motor program is refined, improving error detection or correction mechanisms, reducing movement variability. Finally, movements become highly automatized, skilled, and consistent, and the motor program is now relatively permanent [32].

The phenomenon underlying motor learning is mostly because of neural plasticity [33]. A review of this topic [33] indicates that motor learning takes place with active practice of a skill and that this activity-dependent neural plasticity can be induced by both lengthy-extensive and brief-intensive practice. The literature supports the benefit of augmented feedback for motor learning. In particular, sonification for correct movement sequences has been shown to enhance motor learning in elite athletes [34] but is less useful for novices who have no idea of the correct movement. Walking is a natural way to get about [35], and as older persons are not novices to walking but have lost the expertise with age, their walking pattern should respond to auditory feedback. This type of *positive* feedback has been shown effective in the short term to improve gait pattern in people poststroke [36]. It is superior to auditory alarms signaling incorrect movements as feedback because good movement is more motivating [34].

This solution to poor gait is unique in that there is positive reinforcement, in real time, which stimulates motor learning of correct gait. The Heel2Toe sensor provides information in real time, in other words, knowledge of performance and not just knowledge of after-the-fact results, which is provided by most other technologies in the field today. This is a completely novel and original approach to gait enhancement. There have been other approaches to monitor step counts, but these have not attempted to improve gait quality. The review of the literature conducted by our team did not find any study focusing on feedback related to gait quality and ankle kinematics.

Finally, debriefing interviews suggested readiness of seniors to adopt technology as long as it is simple and user friendly. This project is timely and relevant to increasing the proportion of older population and builds upon the potential of technology to stimulate innovation, thereby advancing Canadian economic and social development. An increasing proportion of older adults use smartphones [37,38], and this proportion is likely to increase as technologically savvy cohorts age.

This sensor could be on the foot of every person who needs to maintain or improve optimal gait. By formally practicing gait improvement with positive auditory feedback, people could develop the habit of walking better leading to walking more often and for longer.

Through the use of the Heel2Toe device, every step becomes therapeutic, engaging large muscle groups, which improves peripheral and core muscle strength and through this improves balance, allows the person to walk at a faster pace. Our data also support changes to gait consistency (lower coefficient of variation with training), making walking more rhythmical, which, in the long run, is more sustainable [35].

The sensor is in development, and refinements to the algorithm will be made, such as to provide different thresholds for the feedback to occur (low, medium, and high AV). An instructional manual and video are in production to optimize the participants’ capacity to use the Heel2Toe sensor. The plan is to develop a full-scale trial, now that there are some data that people can change their gait with the device.

### Limitations

This was a very small study focusing on proof-of-concept only. On the basis of the results that short-term intensive training with positive auditory feedback produced changes in gait quality, a full pilot study is warranted including the second motor learning phase and longer-term practice to estimate sustainability.

### Implications and Conclusions

The results of this study have future implications in exploring the neural basis of auditory-motor synchronization during walking, application of motor learning principles to enhance walking performance, and technology design of wearable sensors for older adults. Understanding the neural basis of auditory motor synchronization will help design interventions to use auditory feedback to improve walking symmetry. The application of motor learning principles to enhance walking performance based on movement-generated auditory feedback and long-term effects on skill acquisition is an area yet to be explored. Debriefing interviews conducted after the intervention concluded that an optimal wearable device for seniors needs to be simple and easy to use, provide real-time meaningful feedback, have a software program that requires minimal preprocessing (zero effort) before use, and have the option for technical support or supervision from a rehabilitation professional [39].

## Abbreviations

AV: angular velocity
LEFS: lower extremity function scale
